# Characterization of Scale Deposits in a Drinking Water Network in a Semi-Arid Region

**DOI:** 10.3390/ijerph19063257

**Published:** 2022-03-10

**Authors:** Pedro Padilla González, Carlos Bautista-Capetillo, Antonio Ruiz-Canales, Julián González-Trinidad, Hugo Enrique Júnez-Ferreira, Ada Rebeca Contreras Rodríguez, Cruz Octavio Robles Rovelo

**Affiliations:** 1 Doctorado en Ciencias de la Ingeniería, Campus UAZ Siglo XXI, Universidad Autónoma de Zacatecas, Carretera Zacatecas-Guadalajara Km. 6, Ejido La Escondida, Zacatecas 98160, Mexico; pad.pa92@gmail.com (P.P.G.); jgonza@uaz.edu.mx (J.G.-T.); hejunez@uaz.edu.mx (H.E.J.-F.); 2 Doctorado en Agricultura, Recursos y Tecnologías Agroambientales y Alimentarios, Miguel Hernández de Elche—Campus de Orihuela, 03202 Orihuela, Spain; aruizcanales@gmail.com; 3 Licenciatura en Ciencia y Tecnología del Agua LUMAT, Universidad Autónoma de Zacatecas, Carretera Zacatecas-Guadalajara Km. 6, Ejido La Escondida, Zacatecas 98160, Mexico; octavio.robles@uaz.edu.mx

**Keywords:** groundwater, drinking water, quality and quantity, scale deposits

## Abstract

The quantity and quality of the supply of fresh water to households, commercial areas, small industries, green spaces irrigation and public and private institutions in large cities face challenges from the supply sources availability and suitable distribution network performance to the full satisfaction of the established drinking water guidelines. In Mexico, the main source of water comes from groundwater. Most of the Mexican aquifers are located in arid and semi-arid weather conditions. The groundwater’s physical–chemical properties are closely related to geology. This study was carried out at the north-central part of the country in which igneous and sedimentary rocks predominate, with high calcium carbonate (CaCO_3_) concentrations. The accumulation of CaCO_3_ in the pipelines is also known as scale deposit that decreases the fluid flow, causing a deficiency in the water supply. The main objectives of this study were determining the physical–chemical groundwater parameters and saturation indexes injected into the drinking water networks and characterizing the scale deposits by scanning electron microscopy (SEM) and X-ray diffraction (XRD). The results indicate that the scale deposits are mainly calcium carbonate and silica oxide crystals, caused by the water aggressiveness according to the saturation indexes and the lack of control over the saturation pH.

## 1. Introduction

Ensuring drinking water supply is vital for human health and consumption [1]. The main source of water to supply the different human demands and develop its different activities comes from groundwater, therefore it is necessary to continue researching the evolution of its quality and quantity. Arid and semi-arid regions face the most challenges in meeting the demand for water for urban public use, irrigation, industry and landscape conservation [2]. Every day, this resource becomes more scarce, while the population grows, which generates a greater demand for water to satisfy its needs, coupled with the fact that natural resources are limited due to global warming, which has caused a greater spatio-temporal variation of the rainfall, in addition to the increase in industrial activities, and the contamination of water resources, generating a deterioration in living standards [3,4]. New alternatives should be considered as sources of water, such as treated wastewater, desalinated water, and harvested rainwater [5,6].

The use of water by humankind to meet its needs has evolved from using ancestral methods, in which water was used directly from the source without any treatment, to the very elaborate ones we use today, which allow the capture of hydric resources and for water to be conducted from very distant sources to households using the driving force in different ways [7]. Nevertheless, nowadays, access to clean drinking water has become a great worldwide challenge [8,9].

Those responsible for supplying water to large cities in Mexico are called water agencies or operating agencies; they provide drinking water and sanitation services to the population and households, always complying with international or national standards of water for human consumption [10].

The piping and distribution networks for drinking water from the source of supply (groundwater or surface water) to households are composed of main and secondary pipes to the household tap, whose diameters depend on the volume of water to be supplied to the network to meet the daily demand for water. The materials used for its installation are polyvinyl chloride (P.V.C), high-density polyethylene (HDPE) and ductile iron (DI). Environmental aspects must be considered in the installation process [11]. Most water operating agencies carry out essential disinfection in drinking water purification processes to protect human health from water-borne diseases (amoebiasis, giardiasis, typhoid fever, dysentery, leptospirosis, cholera, etc.) [12,13,14].

Groundwater disinfection depends on its physical-chemical parameters, which must meet quality guidelines for human consumption, established by the World Health Organization (WHO) or the local standard. The Langelier saturation index (LSI), the aggressiveness index (AI), the Ryznar stability index (RSI), the Puckorius index (PI) and the Larson-Skold index (LS), are obtained from the ions related to hardness (Ca^2+^, Mg^2+^), in addition to other cations (Fe^2+^, Na^+^, K^+^) and different amounts of anions (Cl^−^, SO_4_^2−^, NO_3_^−^, HCO_3_^−^) [15]. These indexes are indicators of groundwater quality and could be used to illustrate the tendency of the groundwater to generate precipitates in the drinking water pipelines, in the same manner, the Puckorius scaling (PSI) and Larson–Skold (LS) indexes allow to estimate the corrosive degree of groundwater.

The quality and safety of drinking water are monitored by water agencies; however, they face the problem of mineral sediment accumulation in the pipes, causing a decrease in the operating efficiency, i.e., a decrease in internal diameter of the pipes, a decrease in the flow of the fluid and above all a possible change in water quality [16]. Generally, when 90 percent of the pipe capacity has been reduced, it is replaced by a new one, which means a large economic investment for the operating agency, since this procedure continues to be repeated over a 5-to-10 year period [16,17].

The nature of the incrustations, or scale deposits, depends on the water circulating through the distribution networks. For example, groundwater quality is significantly influenced by geological formation and anthropogenic activities [18,19,20,21].

The foregoing becomes a scientific challenge, since the mechanism that causes this phenomenon must be understood, although several investigations have been carried out, there are still several questions to be resolved and especially considering the space-time variability of the behavior of mineral deposits, in which we focus on this research [22].

Some authors report that the scale deposits formation inside the pipelines is due to the precipitation of poorly soluble salts such as calcium carbonate, silicates, phosphates, iron, among others, forming different layers on the walls of the water supply pipes, reducing the useful life of these or modifying the water quality that is delivered to households [23,24]. The scale deposits were characterized by X-ray diffraction (XRD) and scanning electron microscopy (SEM) [25,26]. Based on this, the aims of this study are (*i*) to determine the physical–chemical parameters and the saturation index of the groundwater in the drinking water network in a semi-arid region, and (*ii*) to characterize the scale deposits in the water distribution pipeline to the household, through X-ray diffraction (XRD) and scanning electron microscopy (SEM).

## 2. Materials and Methods

### 2.1. Study Area

Zacatecas state is located in the north-central part of Mexico, between the geographical coordinates 25°09′ north, 21°04′ south of north latitude, and to the east 100°49′, with an average altitude of 2500 m above sea level. The study area is characterized by having a variety of climates; however, arid and semi-arid prevail in most of its territory, with stunted rains ranging from 50 to 450 mm annually, although in the southeast of the state, there is an average annual rainfall of 700 mm. The temperature ranges from 12 to 25 °C, the lowest in winter and the highest in summer. Approximately 55 percent of the population lives in urban cities, where there is the least rainfall and the highest demand for water for urban public use. Due to the geographical and climatological characteristics of the state of Zacatecas, surface waters are scarce with small reservoirs distributed along the state. Therefore, groundwater is the main source of public, industrial, and agricultural supply.

**Figure 1:**
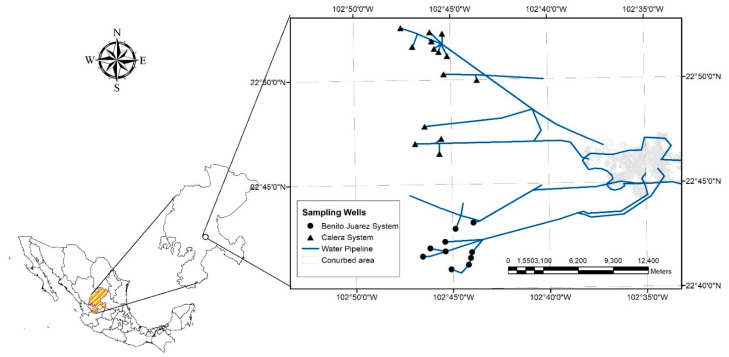
Water distribution system of the Zacatecas conurbation area.

The intermunicipal board of potable water and sewerage (JIAPAZ) is responsible for providing the potable water endowment service to the conurbation area of Zacatecas-Guadalupe, with approximately 450,000 inhabitants. Because the main source of water comes from groundwater, JIAPAZ has drilled wells in some aquifers near the city since 1938, called the Zacatecana system. As the population increased, the volume of water required has been extracted from the Calera aquifer, which is characterized by calcite, dolomite, and pyrite rocks found predominantly in semi-arid regions and are known for their composition of calcium and bicarbonate ions. The water hardness depends on the dissolved ions, the more ions dissolved from rocks and from the soil, the harder the water will be. Very hard water can be expected from areas composed of limestone. The water that gushes into areas made up of other rocks and small amounts of disintegrated silicates is considered soft water, such as rainwater [23]. The Guadalupe-Bañuelos system began its activities in 1982, and the most recent, the Benito Juárez, emerged in 1991. In addition, there are three subsystems: the Morelos, which is part of the Calera system; the Hormigueros, which is part of the Bañuelos-San Ramón system, and the Pimienta, created in 2000–2001, with three wells in the Benito Juárez system and one in the Calera system (Figure 1). The catchment network is made up of 52 wells and several water pumps, with an average flow of 1300 L per second.

### 2.2. Drink Water Samples and Saturation Index

From the Calera and Benito Juárez systems, ten groundwater wells that feed the water conduction and distribution network were monitored from 2015 to 2020. The methodology proposed by APHA was used to determine pH, Ca^2+^, Mg^2+^, alkalinity, temperature, total dissolved solids (TSD), HCO_3_^−^, SO_4_^2−^, Na^+^, K^+^, electrical conductivity (CE), flour, and chlorides, which are indicators that contribute to the corrosion and scale formation in water conduction pipes [27]. Several investigations continue to use the Langeilier saturation (LSI), Ryznar stability (RSI), Puckorius scaling (PSI), Larson–Skold (LS), and Aggressive (AI) indexes to estimate the tendency of calcium carbonate to precipitate in a water conduction or distribution pipe. The equation, the variables and the confidence intervals used to calculate the saturation indexes are presented in Table 1 (according to EPA) [28]. In this study, these indexes were used to determine the condition of the water that is injected into the drinking water network and indicate its possible relationship with the precipitates in the pipeline.

Some authors [29,30] indicate that using saturation indexes such as the Langelier saturation index (LSI) presents several limitations in defining the degree of corrosion of a water source, since it does not consider some parameters that can contribute to the formation of salt such as sodium, suggesting that to determine the degree of corrosion of the water, the dissolved inorganic carbon (DIC) should be considered. The DIC produces an estimate of the amount of total carbonates in the form of carbon dioxide gas (CO_2_), bicarbonate ion (HCO_3_^−^), and carbonate ion (CO_3_^2−^). Generally, the dissolved inorganic DIC is present in groundwater because of the interaction of CO_2_ and H_2_O with rocks. The DIC concentration ranges from less than 20 µM in acidic soft waters to more than 5000 µM in highly alkaline hard waters but ranges from 100 to 1000 µM in most systems. Bicarbonate and carbonate are the main buffers in most natural waters and account for most of the acid-neutralizing capacity (ANC; also called alkalinity) [31].

The following equation shows how to estimate the total amount of inorganic carbon:

(1) $$DIC=CO_{2}+H_{2}CO_{3}+CO_{3}^{2-}+HCO_{3}^{-}\;\;\;$$

The ideal is to determine the DIC of the water directly, but sometimes the equipment is not available; therefore, the US Environmental Protection Agency (US EPA 2003) recommends an indirect estimate (Equation (1)), which, after analysis in more than 1300 wells, found a relationship between pH and alkalinity to estimate the DIC based on these parameters. In this study, the function of pH and alkalinity was used to determine the DIC (adapted from US EPA, 2003).

### 2.3. Pipes Sampling

**Figure 2:**
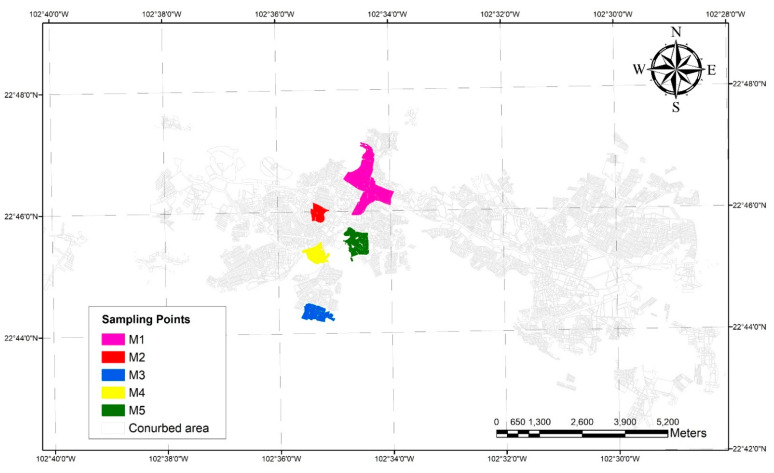
Sampling points in the conurbation area.

The samples analyzed were provided by the company responsible for supplying the water, JIAPAZ. These samples were obtained from different points of the city of Zacatecas’s drinking water pipe network. In Figure 2, the sampling points are marked. The five pipe samples are a combination of 10 wells: four of the samples are PVC pipes and only one is asbestos (first sample). In the 1950s and 1960s, the first pipes used were made of asbestos; however, with the scientific reports related to the negative effects on human health, they were replaced with PVC in the 1970s [32]. In this study, samples of scale deposits from the asbestos pipes were taken to show that the scale deposits are present in both PVC and asbestos materials. Pipe samples 1 and 2 (classified as M1 and M2) correspond to the Calera system which is integrated by 5 wells (classified as C1 to C5). On the other hand, pipe samples 3, 4 and 5 (M3, M4, and M5) carried water from the Benito Juárez system particularly from the B1 to B5 wells. The sampling was carried out in the conurbation area of the city.

**Figure 3:**
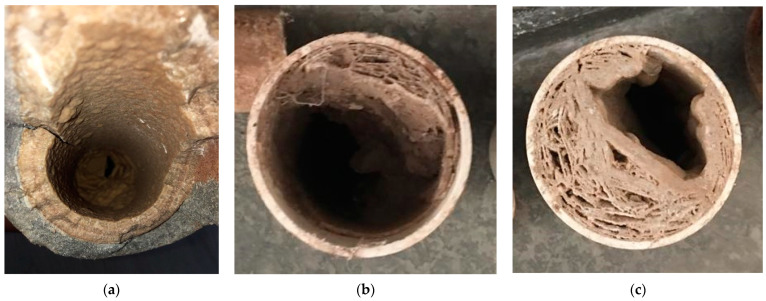
Asbestos (**a**) and PVC (**b**,**c**) sample pipes collected from different points along the Zacatecas-México distribution network.

Figure 3 shows some images of the pipes where the scale precipitation is observed, to the extent of significantly interrupting the passage of the liquid in the distribution networks (Figure 3c) in the city of Zacatecas.

### 2.4. Pretreatment Process

The drinking water network consists of several deep wells drilled at approximately 300 m, while the water level is located at 80 m. Subsequently, there is a 6-inch pipe to deposit the water in a pumping system, however, before reaching this point, no treatment is given to the groundwater [33,34]. The water is then conducted to a distribution tank where it is chlorinated with sodium hypochlorite (NaOCl), in order to comply with the recommendations established by the WHO [35,36], the United States Environmental Protection Agency (USEPA) [37] and the Mexican Official Standards (NOM’s) [38,39]. The maximum value of residual free chlorine that applies to drinking water is 5 mg/L [35]. Hardness and other elements such as fluorine, arsenic or iron in groundwater are not treated by any specific method.

**Figure 4:**
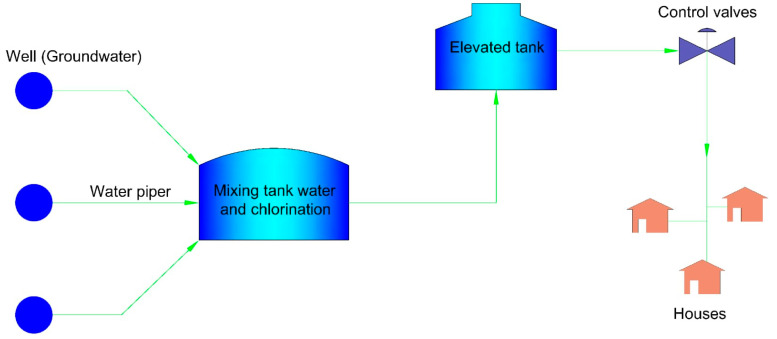
Distribution system and water chlorination treatment.

Figure 4 shows the distribution system, the pipelines are affected by the scale deposits located after the mixing tank water and chlorination.

### 2.5. Characterization of Precipitates

#### 2.5.1. X-ray Diffraction (XRD)

The X-ray diffraction technique has been widely used in the characterization of materials that meet the condition of having a defined crystallographic structure, since the information obtained from the interaction between X-rays and crystals is based on the diffraction produced by a set of atoms in an ordered arrangement. X-ray diffraction by crystalline materials is a coherent scattering process. This radiation originates from the impact of the incident photons against the electrons bound to the atoms. Each irradiated atom of the material individually scatters rays in all directions; these are in phase and produce constructive interference of waves, and it is in these directions that intensity maxima are observed in diffraction [40].

The XRD analysis was conducted using the powder of the samples, and to do so, small samples were extracted and dried in a thermostatized oven for 48 h at 40 ° C. Then, the samples were crushed using an agate mortar until approximately 98% of the sample passed a No. 325 mesh [41]. Once the powder was obtained, it was placed in a special capsule for later analysis. XRD analyses were carried out on a Bruker ECO D8 Advance diffractometer.

To analyze the phase transformation, the XRD technique was conducted by means of Cu-Kα radiation on a D8 Advanced-ECO Bruker™ diffractometer at room temperature. A continuous scan mode in a coupled two-theta-theta mode, in the two-theta range of 10–90 degrees with a step size of 0.01°, was set up in the instrument.

#### 2.5.2. Scanning Electron Microscopy (SEM)

Scanning electron microscopy (SEM) is a topographic, structural, and compositional analysis technique. In general terms, SEM equipment can take an “image” of the sample, although what is really detected is the response of the material to the impact of an electron beam [26].

A small amount of deposited scale was taken from the pipe walls before the start of the experiment and analyzed using SEM. Preliminary preparation of the samples was required, with a palladium coating to compensate for its insulating nature, allowing electrical charges to be evacuated during observation and increasing the conductivity of the sample. For this observation, the SEM-Hitachi S-3500N scanning electron microscope (Ibaraki, Japan) was used.

## 3. Results and Discussion

Understanding the evolution of the water quality in the subsoil allows identifying possible elements that are considered hazardous and, therefore, regulated by the norms established for human consumption in the different countries. In semi-arid regions, groundwater becomes the main source of supply to satisfy the needs of the population, since rainfall is scarce, the sources of surface water also become scarce. The water–rock interaction continues to be a scientific challenge since this phenomenon contributes to the understanding of the evolution of the quantity and quality of water over time. In addition to explaining the origin of the chemical compounds in the water used by the population in its different productive activities, it helps to avoid possible effects on human health.

### 3.1. Water Chemistry Characterization

The focus of the study was to identify the origins of the scale deposits that occur in the distribution network of drinking water, which reduce the useful life of the pipe and generate problems in the delivery of water to the population. The analyzed chemical and physical properties of drinking water for the wells of both aquifers, the Calera and Benito Juárez systems are presented in Table 2.

The temperature from the Calera system varied from 23 to 26 °C, which indicates that the water has a brief run through the geological environment (calcites) or has been mixed [42]. For the Benito Juárez system, the temperature range was from 23 to 30 °C; this last value indicates that the water has had a longer run in the geological system. The pH is the parameter that directly influences the precipitation of calcium, stimulating the formation of scales in pipes [43]. The underground water injected into the urban network presents pH values from 7.7 to 8.4, according to the EPA classification, the water is considered alkaline, and some authors report that these pH ranges contribute to the precipitation, especially of calcium carbonate (CaCO_3_) [30,44].

Regarding electrical conductivity, the average value of 407 μmhos/cm is below the guidelines recommended by the WHO and the Mexican standard NOM-SSA-1997, modified in 2000 [38], which is 700 μmhos/cm. On the other hand, some authors indicate that a small electrical conductivity in groundwater can be attributed to young water, also called local flow, which can be reinforced with the evolution of cations and anions. For the latter, this would be HCO_3_^−^ > SO_4_^2−^ > Cl^−^ > Ca^2+^ > Mg^2+^ > Na^+^ [45], this coupled with reports of low TDS. In this study, they ranged from 185 to 230 mg/L, and these data are in accordance with categorizing the water as young.

According to the Mexican standard NOM-SSA-1997, modified in 2000 (DOF), the water of all the wells complies with the concentration standards regarding chlorine, fluorite, TDS, calcium carbonate, and pH recommended for urban public use. Table 3 shows the water quality based on the measured and standard values for both wells systems, Calera and Benito Juárez, with respect to the Mexican standard.

The characteristics of the water comply with the Mexican standard, that is, it boasts good quality; however, scale deposits are present in the pipes according to the identification of the ions present, calcium carbonate and silica oxide, which are also present in the chemical composition of the groundwater that is injected into the drinking water network. Taking the chemical parameters into account, it is necessary to evaluate its behavior through saturation indexes and DIC.

The LSI index varied from −0.005 to 0.76, the negative value of C4 indicates that the water in C4 will dissolve CaCO_3_; on the other hand, the index values of C1, C2, C3 and C5, in the Calera system, suggest that are supersaturated waters. Undersaturated water tends to remove existing calcium carbonate protective coatings in pipelines and equipment; however, scale deposits may occur with CaCO_3_ supersaturated water. The RSI from 7 to 7.9 classified the water as undersaturated, therefore it tends to dissolve solid CaCO_3_. The PSI index ranged from 6.9 to 7.8, placing it as aggressive water (significant corrosion); meanwhile, the behavior of LS ranged from 0.1 to 0.15, indicating that chlorides and sulfates probably will not interfere with natural film formation. Lastly, the AI index showed a value of 10 to 11, indicating moderately aggressive water, for the source of the water supply to the network called La Joya, which is part of the Calera system (Table 4). All LSI values for the Benito Juárez system are below zero indicating undersaturated water. In the case of the RSI index, the values (7.4–8.5) indicate that the condition of the water is also undersaturated. On the other hand, the PSI index having values above 7 suggests that the water is likely to dissolve scale. The values of the LS index for both, the Calera and Benito Juárez systems, are similar so it is expected that there will be no interference from chlorides and sulfates in the B1 to B5 wells. Although the values of the aggressiveness index in B1 and B2 are the lowest with respect to the other wells, they are still considered as very aggressive water. Overall the Benito Juárez underground water well system presented a similar behavior to the Calera system, which can be attributed to the fact that the water passed through a similar geological environment.

As shown in Figure 4, the drinking water system from the different wells mixes in the mixing tank, which causes a change in the LSI of 0.38 due to the concentration of the calcium and carbonate ions, although the water remains saturated, a decrease in saturation is seen, helping the period of encrustation of salt deposits in the pipe which occurs over long periods of time. The RSI calculated after the wells are mixed is 7.6, indicating that the water remains undersaturated; the same behavior is shown in the LS and AI index.

However, when using the Langelier-based index, improved by Carrier (1965), the water is considered scale-forming but not corrosive. Nevertheless, as indicated above, this index has some deficiencies, since it does not consider other parameters of the chemical composition of water such as phosphates and silicates, elements that are part of the groundwater that is injected into the network monitored in this study. It was found that the Langelier-modified index by Carrier varied from 22 to 70 mg/L, and hence it must be considered when calculating the degree of saturation of the water. Therefore, other indexes also need to be considered, such as the DIC (dissolved inorganic carbon) (Table 5).

Larsen [46] suggests that the relation Ca^2+^/HCO_3_^−^ is an important factor in the precipitation of CaCO_3_. The Pearson coefficient (0.18) was used to demonstrate the relation between ions; according to Rosales-Rivera [47], the value obtained is low, therefore, the precipitation of the scale deposits is favored.

Armendáriz et al. [48] suggested that the precipitation of the calcite is related to the concentration of Mg^2+^ ions, if the Ca^2+^/Mg^2+^ > 4 > 4 precipitation of calcite is favorable. In the drinking water distribution network, the concentration of calcium varied from 18 to 58 mg/L, while the magnesium varied from 4.47 to 11.98 mg/L; obtaining a mean Ca^2+^/Mg^2+^ relation of 8 (Table 5). Some studies report that calcium can prevent the system’s ability to raise the pH, which causes the scale incrustations in the pipes of the conduction network, reducing the diameter and the hydraulic load of the pipe as well as the cost of distribution, limiting the service to the users of drinking water [49]. Generally, when the scale incrustations are characterized, calcium does not appear individually, but rather in combination with other elements, in the form of calcium carbonate. The EPA recommends that before applying any treatment the drinking water utilities should monitor the pH saturation, since maintaining a pH below 8 decreases the possibility of calcium carbonate scale; however, it also suggests that the behavior of trace metals and phosphates should be monitored.

According to the DIC, calculated by the method proposed by the EPA, water of medium solubility indicates that it can favor the calcium carbonate precipitate, added to a low saturation pH. Therefore, in order to apply any treatment for the removal of the carbonate, it is important to first stabilize the saturation pH. However, in the characterization of the scale deposit, it was found to be associated with other components, such as silicon oxide, which can generate greater difficulty in removing scale deposits. On the other hand, chlorinating the water with sodium hypochlorite for the disinfection of the water, thus complying with the standards of the World Health Organization and the Mexican standards, allows or contributes to raising the pH, which causes the pH of saturation to be more unstable.

### 3.2. Scale Composition

**Figure 5:**
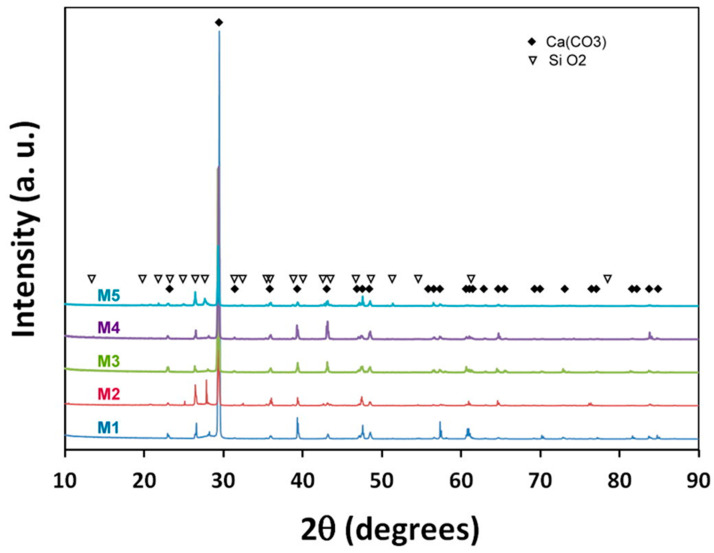
X-ray diffraction pattern confirming the presence of CaCO_3_ (black diamond) and SiO_2_ (triangle) phase in all samples.

From the groundwater chemical characterization, we can see that calcium and bicarbonate are present, which suggests the presence of calcium carbonate. CaCO_3_ can exist in three different crystal forms (calcite, aragonite and vaterite) as well as an amorphous phase [29]. All specimens resulted in CaCO_3_ and SiO_2_ phases, as illustrated by the diffraction patterns in Figure 5. A rhombohedral crystal structure corresponding to a space group of R-3c(167) was identified for the CaCO_3_ phase, whereas a cubic crystal structure corresponding to a space group of Pm-en(223) was obtained for SiO_2_ phase. All the wells sampled presented silica concentrations between 29 and 66 mg/L, which could explain the presence of the oxide.

It also can be seen from the X-ray diffractograms that the peak maxima are observed at approximately 2θ = 29.39, corresponding to the crystallographic phase of calcite [43].

According to a semi-quantification procedure, the higher volume fraction corresponded to CaCO_3_ (i.e., above 66 pct. for samples M2 and M5, and 91 pct. for the remaining samples). The semi-quantification results are listed in Table 6.

**Figure 6:**
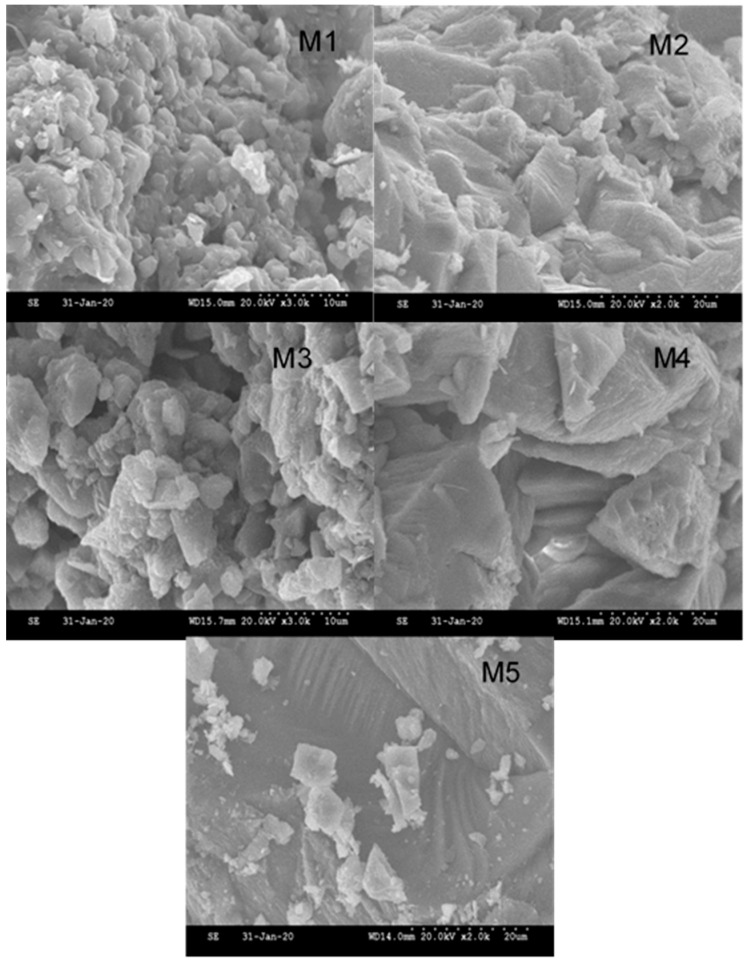
SEM photographs of crystals grown in asbestos, **M1**, and PVC pipes from **M2** to **M5**.

The samples consist of a mixture of square and hexagonal crystals characterized as calcite, which are very difficult to remove since they strongly adhere to the walls of the pipe. These data coincide with those reported by Moulessehoul et al. [50], based on an experiment that characterized the precipitates of some pipes (Figure 6). SEM micrographs of each sample illustrate the details of the crystals and amorphous particles formed in each pipe. A comparison between the different micrographs allows the definition of the crystallization state of the calcite. Several parameters can influence the crystal size, such as agitation, pressure, ion concentration, temperature, and pH.

The micrograph of a general view of the precipitated sample reveals a strong crystallization, the magnification of which shows the presence of rectangular facies and elongated rods similar to those of struvite, which were reported by Bouropoulos et al. [51]. Due to the presence of silica in the groundwater, the crystallization of the calcium carbonate may be affected, having more than one type of structure. It can be said that they have crystallized in a polyform way, and it is common to present this type of crystallization since the changes in both temperature and pressure are factors that influence crystallization. The crystals do not particularly belong to this mineral type due to its crystallized form having more than one type of structure [52].

## 4. Conclusions

The role of calcite in understanding the crystallization process has been highlighted. The specific and original contribution of this study shows that the control of the reaction at a specific pH contributes strongly to the precipitation conditions, in terms of the amount of silicon and calcium (Zacatecas, Mexico).

The results obtained evidence of the particular interest in understanding the physicochemical phenomena involved in the crystallization of the different minerals in the pipes.

The approach adopted was based on monitoring the crystallization within the pipes, based on the influence of time on these pipes and the precipitated amount of minerals, of which calcite has a strong adherence to the pipe walls. These observations are in agreement with the analysis of the morphological characteristics of the scale deposits obtained. From the SEM analysis, it can be concluded that the morphology of the scale deposits is polymorphous due to the nature of the reaction itself and considering that the scale samples were obtained from a real medium. In addition, the analysis by X-ray diffraction allowed the determination of not only the presence of CaCO_3_, but also the crystallographic form, calcite. Furthermore, the presence of SiO_2_ was also identified as part of the composition of the scale deposit.

Numerous research works have been carried out with water that has been manipulated so that it contains certain physical-chemical characteristics that favor the formation of scale deposits (CaCO_3_); however, in this study, all the measurements involved in the precipitation of CaCO_3_, such as the ions concentration (Ca^2+^, Mg^2+^, HCO_3_^−^, etc.), SiO concentration, pH and electric conductivity, were determined from real world aquifers and water supply networks in a semi-arid Mexican region.

Future research should be focused on the continued monitoring of the evolution of calcium scales and identifying if the water purification process has any impact on the formation of scale deposits by monitoring the water system before it enters the urban network, in order to obtain a better understanding of the process and, in doing so, be able to recommend some actions to prevent scaling and increase the lifespan of the pipelines.

## Figures and Tables

**Table 1:** Saturation indexes, equations, and criteria for categorizing the stability of the water.

Index	Equation	Index Value	Water Condition
Langelier saturation (LSI)	$LSI=pH-pH_{s}$ If the pH≤9.3 then use $pH_{s}=A+B-\log\;\left[Ca^{2+}\right]-\log\left[alk\right]$ Else, pH>9.3 then use $pH_{s}=\left(9.3+A+B\right)-\left(C+D\right)$ $A=\frac{\log\left[TDS\right]-1}{10}$ B=−1.12[log (°C+273)]+34.55 $C=\log\;\left[Ca^{2+}+CaCO_{3}\right]-0.4$ $D=\log\;\left[\mathrm{alkalinity}\;\mathrm{as}\;CaCO_{3}\right]$	LSI > 0 LSI = 0 LSI < 0	Super saturated, tends to precipitate CaCO_3_ Saturated, CaCO_3_ is in equilibrium Undersaturated, tends to dissolve solid CaCO_3_
Ryznar stability (RSI)	$RSI=2\left(pH_{s}\right)-pH$	RSI < 6 6 < RSI < 7 RSI > 7	Super saturated, tends to precipitate CaCO_3_ Saturated, CaCO_3_ is in equilibrium Undersaturated, tends to dissolve solid CaCO_3_
Puckorius scaling (PSI)	$PSI=2\left(pH_{s}\right)-pH_{eq}$ $pH_{s}=1.465+\log\left[alk\right]+4.54$ $pH_{eq}=1.465\;x\log\left[alk\right]+4.54$	PSI < 6 PSI > 7	Scaling is unlikely to occur Likely to dissolve scale
Larson–Skold (LS)	$LS=\left(Cl^{-}+SO_{4}^{2-}\right)/\left(HCO_{3}^{-}\right)+CO_{3}^{2-})$	LS < 0.8 0.8 < LS < 1.2 LS > 1.2	Chloride and sulfate are unlikely to interfere with the formation of protecting film Corrosion rates may be higher than expected High rates of localized corrosion may be expected
Aggressive (AI)	AI=pH+log[(alk)(H)]	AI > 12 10 < AI < 12 AI < 10	Non-aggressive Moderately aggressive Very aggressive

**Table 2:** Ion concentrations of the water samples.

Well Identifier	pH	Temp (°C)	CE (μmhos/cm)	Na^+^	Ca^2+^	HCO_3_^−^	SO_4_^2−^	Cl^−^	Alkalinity	TDS	Hardness
				(mg/L)							
C1	7.7	23	375	32	58	256	30	8	210	185	170
C2	7.7	26	375	35	58	276	19	8	226	193	129
C3	8.4	26	435	51	37	217	32	12	210	220	155
C4	7.8	26	380	34	35	167	10	14	138	186	105
C5	8.1	26	470	70	18	235	26	20	193	230	56
B1	6.7	23	439	45	48	252	12	19	207	215	126
B2	6.8	24	441	53	40	239	4	17	196	216	139
B3	7.9	25	447	35	48	200	24	20	194	219	143
B4	8.0	28	498	54	40	258	26	21	211	244	144
B5	7.9	30	508	75	33	224	30	20	184	248	170

**Table 3:** Water quality classification based on the Mexican standard and the measured values.

Parameter	Mean Values Measured from the Groundwater Wells	Standard Values According to the NOM-SSA-1997	Water Quality Classification
Hardness (CaCO_3_) *	130.0	500	In range
Chloride (Cl^−^) *	15.0	250.0	In range
Fluoride (F^−^) *	1.2	1.5	In range
pH	7.7	6.5–8.5	In range
Sodium *	46.0	200.0	In range
Total dissolved solids *	212.0	1000.0	In range
Sulfate (SO_4_^2−^) *	20.0	400.0	In range

* Units in mg/L.

**Table 4:** Saturation indexes for the groundwater extraction wells.

Well Identifier	LSI	RSI	PSI	LS	AI
C1	0.31	7.3	7.1	0.15	10.0
C2	0.39	7.1	6.9	0.10	10.0
C3	0.76	7.0	6.9	0.10	11.0
C4	−0.005	7.8	7.0	0.15	10.0
C5	0.016	7.9	7.8	0.15	10.5
B1	−0.67	8.4	7.2	0.12	9.0
B2	−0.66	8.5	7.4	0.10	9.0
B3	0.63	7.0	7.1	0.22	10.0
B4	0.62	7.1	7.1	0.18	10.0
B5	0.40	7.4	7.4	0.22	10.0

LSI: Langelier; RSI: Ryznar stability index; PSI: Puckorius scaling index; LS: Larson–Skold index; AI: Aggressive index.

**Table 5:** DIC estimation and pH saturation.

Well Identifier	pH	Temp (°C)	Alkalinity (mg/L)	DIC (mg/L)	Saturation pH	Ca^2+^ (mg/L)	Mg^2+^ (mg/L)	SiO (mg/L)
C1	7.7	23.3	210	49	7.6	58	11.98	54
C2	7.7	26	226	55	8.0	58	2.70	52
C3	8.4	26	210	48	7.8	37	4.5	66
C4	7.8	26	138	37	8.0	35	4.2	38
C5	8.1	26.4	193	49	8.2	18	3.6	58
B1	6.7	23	207	64	7.8	48	5.51	64
B2	6.8	24	196	64	7.8	40	2.47	29
B3	7.9	25	194	49	7.6	48	5.93	43
B4	8.0	28	211	55	7.8	40	3.47	38
B5	7.9	30	184	35	8.0	33	7.57	49

**Table 6:** The volume fraction of the two identified phases in all specimens as per a semi-quantification procedure.

Sample					
Phase	M1	M2	M3	M4	M5
CaCO_3_	93.6	66.6	94.9	91.4	69.5
SiO_2_	6.4	33.4	5.1	8.6	30.5

## Data Availability

The data presented in this study are available in the article.
